# Soluble urokinase plasminogen activator receptor (suPAR) in bronchoalveolar fluid and blood in critically ill patients—a prospective cohort study

**DOI:** 10.1007/s15010-023-02127-3

**Published:** 2023-11-16

**Authors:** Alexander C. Reisinger, Stefan Hatzl, Juergen Prattes, Gerald Hackl, Gernot Schilcher, Florian Eisner, Tobias Niedrist, Reinhard Raggam, Robert Krause, Philipp Eller

**Affiliations:** 1 Intensive Care Unit, Department of Internal Medicine, Medical University of Graz, Auenbruggerplatz 15, 8036 Graz, Austria; 2 Division of Infectious Diseases, Department of Internal Medicine, Medical University of Graz, Graz, Austria; 3 Clinical Institute of Medical and Chemical Laboratory Diagnostics, Medical University of Graz, Graz, Austria; 4 Division of Angiology, Department of Internal Medicine, Medical University of Graz, Graz, Austria

**Keywords:** suPAR, Critical care, ARDS, Fungi, Aspergillosis, Bronchoalveolar lavage

## Abstract

**Introduction:**

Soluble urokinase plasminogen activator receptor (suPAR) is a biologically active protein and increased levels are associated with worse outcomes in critically ill patients. suPAR in bronchoalveolar fluid (BALF) may be helpful to differentiate between types of acute respiratory distress syndrome (ARDS) and may have potential for early detection of fungal infection.

**Methods:**

We prospectively investigated levels of suPAR in BALF and serum in critically ill patients who underwent bronchoscopy for any reason at the ICU of the Department of Internal Medicine, Medical University of Graz, Graz, Austria.

**Results:**

Seventy-five patients were available for analyses. Median age was 60 [25th–75th percentile: 50–69] years, 27% were female, and median SOFA score was 12 [11–14] points. Serum suPAR levels were significantly associated with ICU mortality in univariable logistic regression analysis. There was no correlation between BALF and serum suPAR. Serum suPAR was higher in ARDS patients at 11.2 [8.0–17.2] ng/mL compared to those without ARDS at 7.1 [3.7–10.1] (*p* < 0.001). BALF-suPAR was significantly higher in patients with evidence of fungal lung infection compared to patients without fungal infection both in the general cohort (7.6 [3.2–9.4] vs 2.5 [1.1–5.3], *p* = 0.013) and in the subgroup of ARDS (7.2 [3.1–39.2] vs 2.5 [1.0–5.2], *p* = 0.022). All patients were classified as putative/probable invasive aspergillosis.

**Conclusion:**

We found significant higher levels of serum suPAR in ARDS patients compared to those not fulfilling ARDS criteria. Serum and BALF-suPAR were significantly higher in those patients with evidence for invasive pulmonary aspergillosis. These findings may suggest testing this biomarker for early diagnosis of fungal infection in a greater cohort.

Soluble urokinase plasminogen activator receptor (suPAR) is the dissolved form of the membrane-bound protein uPAR, which is found on endothelial cells, neutrophils, and activated macrophages [1]. suPAR is part of the three-finger fold protein toxin superfamily which has strong signaling capabilities [2, 3]. Circulating suPAR levels in blood are usually low but increase during inflammation and immune system activation. The normal serum levels for healthy individuals are reported to be 2–3 ng/mL [4]. Critically ill patients and patients with sepsis have elevated levels of suPAR, and suPAR is a strong prognostic marker for outcomes. suPAR may also predict the occurrence of acute kidney injury during intensive care unit (ICU) stay [5–7]. In a small retrospective study, suPAR in bronchoalveolar fluid (BALF) was higher in patients with bacterial acute respiratory distress syndrome (ARDS) compared to viral ARDS and controls without active pulmonary disease [8].

Besides bacterial and viral infections, ICU patients are also prone to develop pulmonary fungal infection. Pulmonary fungal infections are a major threat for patients with ARDS but may also affect patients without ARDS on ICU [9–11]. Up to date, there is no data on suPAR levels in BALF in patients with pulmonary fungal infections. Early differentiation of ARDS etiology, however, is of paramount importance to guide preemptive antimicrobial treatment, and consequently improve overall ARDS outcome as ARDS is still associated with high morbidity and mortality. We hypothesized that suPAR may have a role as a semi-quantitative biomarker in BALF and may help to differentiate between underlying pulmonary diseases including the presence of fungal infections.

In this study, we prospectively investigated concentrations of suPAR in BALF and serum in unselected adult critically ill patients who underwent bronchoscopy for any reason at the ICU of the Department of Internal Medicine at the Medical University of Graz, Austria. Exclusion criteria were patients in palliative care or comfort terminal care only, and a decline to participate. BALF and serum samples were obtained simultaneously. Sequential bronchoscopies in patients who underwent more than one bronchoscopy were not investigated. The presence or absence of ARDS was noted, as well as the underlying pathology or infectious causes. ARDS was defined according to the Berlin definition [12]. Evidence of mold infection was noted and classification of pulmonary fungal disease was made according to established consensus definitions [13, 14].

BALF was obtained during routine bronchoscopy by instillation of sterile saline through a fiberoptic bronchoscope. Samples were collected in sterile containers. suPAR was measured in batch with the commercially available suPARnostic® ELISA (ViroGates A/S, Copenhagen, Denmark) after recruitment.

The main endpoint was the investigation of BALF-suPAR for ARDS etiology and mortality. The study protocol was approved by the Institutional Review Board of the Medical University of Graz, Austria (32–472 ex 19/20), registered in the WHO approved German study registry (DRKS00022458) and complied with the Declaration of Helsinki. Written informed consent was obtained from all conscious patients. In comatose non-survivors, the IRB waived the need for informed consent. All statistical analyses were performed with SPSS 27 (SPSS Inc, IBM, Armonk, NY). Continuous variables were summarized as medians [25th–75th percentile], and categorical variables as absolute frequencies (%). Associations between variables were computed with cross-tabulations, Mann–Whitney U tests, and Fisher’s exact tests, as appropriate. Spearman’s rank-based correlation coefficient was used for correlations analyses. Significance level was determined at 0.05.

During the study period from February 2021 to December 2022, 76 consecutive patients were included. One patient withdrew consent; therefore, seventy-five patients were available for analyses. The median age was 60 [25th–75th percentile: 50–69] years, 27% were female, and the median SOFA score at sample recovery was 12 (11–14) points reflecting a high disease severity (Table 1). Median time to sample acquisition after endotracheal intubation was 2.5 [0.5–17.0] h.

**Table 1:** Parameters of the total cohort, and patients with or without ARDS

	Total cohort	Patients with ARDS (*N* = 42, 56%)	Patients without ARDS (*N* = 33, 44%)	*p* value
Age (years)	60 [50–69]	60 [51–65]	61 [49–75]	0.525
Female sex	20 (27%)	16 (38%)	4 (12%)	**0.017**
SOFA score at BALF acquisition	12 [11–14]	12 [11–15]	12 [11–13]	0.515
Vasopressor at BALF acquisition	64 (85%)	36 (86%)	28 (85%)	0.916
RRT at BALF acquisition	4 (5%)	1 (2%)	3 (9%)	0.314
OI (mmHg)	123 [80–239]	88 [72–169]	143 [96–292]	**0.006**
ICU LOS (days)	8.4 [4.4–14.6]	10.7 [4.9–20.4]	7.4 [3.1–11.7]	0.058
ICU mortality	35 (47%)	22 (52%)	13 (39%)	0.352
suPAR in serum (ng/mL)	9.3 [6.3–13.2]	11.2 [8.0–17.2]	7.1 [3.7–10.1]	** < 0.001**
suPAR in BALF (ng/mL)	2.7 [1.2–7.2]	2.7 [1.2–6.0]	2.5 [1.4–7.4]	0.827

Bold *p* values are statistically significant

*ARDS* acute respiratory distress syndrome, *SOFA* sequential organ failure assessment, *BALF* bronchoalveolar lavage fluid, *RRT* renal replacement therapy, *OI* oxygenation index (paO_2_/FiO_2_), *ICU LOS* intensive care unit length of stay, *suPAR* soluble urokinase plasminogen activator receptor

suPAR levels in BALF were median 2.7 [1.2–7.2] ng/mL and serum levels were median 9.3 [6.3–13.2] ng/mL. Serum suPAR levels were significantly associated with ICU mortality in univariable logistic regression analysis (OR per 1 ng/mL increase 1.08 [1.01–1.17], *p* = 0.048). There was no correlation between BALF and serum suPAR levels (Spearman rho = – 0.043, *p* = 0.811). ARDS was present in 42 patients with a median oxygenation index of 88 [72–169] mmHg. There were no significant differences in SOFA score between ARDS patients and other patients (12 (11–15) vs 12 (11–13) points, *p* = 0.515). BALF-suPAR levels were similar in those patients with ARDS compared to those not fulfilling the ARDS definition. However, serum suPAR was higher in ARDS patients at 11.2 [8.0–17.2] ng/mL compared to those without ARDS at 7.1 [3.7–10.1] ng/mL (*p* < 0.001). In those patients with ARDS, suPAR was similar in bacterial compared to viral ARDS both in serum and BALF (p values: 0.822 and 0.869).

Seven patients were diagnosed with pulmonary fungal infection during routine care. BALF-suPAR was significantly higher in patients with evidence of pulmonary aspergillosis compared to patients without signs for fungal lung infection (7.6 [3.2–9.4] vs 2.5 [1.1–5.3] ng/mL, *p* = 0.013; Table 2). All patients were classified as putative/probable invasive aspergillosis. Similar results were found for the subgroup of ARDS patients (7.2 [3.1–39.2] vs 2.5 [1.0–5.2] ng/mL, *p* = 0.022; Table 2).

**Table 2:** Patients with evidence of invasive aspergillosis in the total cohort and subgroup of patients with ARDS

	Total cohort: patients with invasive pulmonary aspergillosis (*N* = 7)	Total cohort: patients without invasive pulmonary aspergillosis (*N* = 68)	*p* value	ARDS cohort: patients with invasive pulmonary aspergillosis (*N* = 6)	ARDS cohort: patients without invasive pulmonary aspergillosis (*N* = 36)	*p* value
Age (years)	60 [58–71]	59 [50–69]	0.799	60 [52–71]	59 [50–65]	0.636
Female sex	3 (43%)	17 (25%)	0.375	3 (50%)	13 (36%)	0.658
SOFA score at BALF acquisition	13 [12–16]	12 [10–13]	0.056	13 [12–17]	12 [10–15]	0.182
Vasopressor at BALF acquisition	5 (71%)	59 (87%)	0.272	5 (83%)	31 (86%)	1.000
RRT at BALF acquisition	0 (0%)	4 (6%)	1.000	0 (0%)	1 (3%)	1.000
OI (mmHg)	91 [68–189]	125 [80–243]	0.623	86 [68–170]	89 [72–178]	0.820
ICU LOS (days)	10.3 [6.2–31.0]	7.9 [3.5–14.5]	0.172	10.1 [5.9–25.5]	11.1 [4.9–19.9]	0.766
ICU mortality	4 (57%)	31 (46%)	0.699	4 (67%)	18 (50%)	0.665
suPAR in serum (ng/mL)	12.3 [10.9–17.6]	9.0 [5.2–11.8]	**0.049**	14.7 [10.1–17.6]	11.1 [7.9–16.4]	0.449
suPAR in BALF (ng/mL)	7.6 [3.2–9.4]	2.5 [1.1–5.3]	**0.013**	7.2 [3.1–39.2]	2.5 [1.0–5.2]	**0.022**

Bold *p* values are statistically significant

Of the presented patients, 3/7 were probable aspergillosis according to COVID-19-associated pulmonary aspergillosis criteria and 4/7 were classified as putative aspergillosis according to diagnosis criteria for pulmonary aspergillosis in critically ill patients. One additional patient had aspergillosis colonization and was not included into the analyses. However, the results were similar in an exploratory analysis including this single additional patient

*ARDS* acute respiratory distress syndrome, *SOFA* sequential organ failure assessment, *BALF* bronchoalveolar lavage fluid, *RRT* renal replacement therapy, *OI* oxygenation index (paO_2_/FiO_2_), *ICU LOS* intensive care unit length of stay, *suPAR* soluble urokinase plasminogen activator receptor

ARDS and invasive fungal infections are devastating diseases in critically ill patients admitted to ICU. Early recognition of underlying causes is necessary to allow for targeted treatment. Diagnosis of fungal infections is difficult and confirmation using biopsy is not feasible in most cases. Hematological malignant diseases are well-known risk factors; however, recently influenza and COVID-19-associated pulmonary aspergillosis have been increasingly recognized [9, 15, 16]. Serum galactomannan lacks sensitivity for pulmonary aspergillosis, while BALF-galactomannan has frequently been used in hematological patients to assess for fungal infections but its role in other patients is not fully delineated. In our study, we found suPAR in serum and in BALF to be higher in patients with evidence of invasive mold infection. Aspergillus species was the only type of mold that was found in our patients. BALF-suPAR was not significantly different between ARDS and non-ARDS patients, but BALF-suPAR in ARDS patients was significantly higher in those patients with evidence for invasive pulmonary aspergillosis compared to those without fungal infection. These findings suggest a potential role of suPAR as a BALF-biomarker for early identification of pulmonary aspergillosis.

A limitation of our study is the absence of healthy controls, but in patients without evidence of lung disease, BALF-suPAR levels were 1.0 [0.5–1.9] ng/mL [8]. Another limitation is the different recovery rate of the amount of saline used during bronchoscopy. As for all BALF biomarkers, standardization is hardly feasible due to differences in the amount of fluid present within bronchial tree independent of the sample volume used. Therefore, a semi-quantitative use of suPAR is a more reasonable approach. However, due to sample size, the optimal cutoff has yet to be determined.

To our knowledge, this study is the first prospective investigation of suPAR levels in serum and BALF in critically ill ICU patients undergoing bronchoscopy. We showed significant higher levels of serum suPAR in ARDS patients compared to those not fulfilling ARDS criteria. Serum and BALF-suPAR were significantly higher in those patients with evidence for invasive pulmonary aspergillosis. These findings may suggest testing this biomarker for early diagnosis of fungal infection in a greater cohort.

## Data Availability

The datasets used and/or analyzed during the current study are available from the corresponding author on reasonable request.
